# Downregulation of lncRNA EPB41L4A-AS1 promotes gastric cancer cell proliferation, migration and invasion

**DOI:** 10.1186/s12876-024-03216-9

**Published:** 2024-04-16

**Authors:** Jiancang Ma, Yingying Feng, Jinkai Xu, Zongyu Li, Jingyue Lai, Hao Guan

**Affiliations:** 1 Department of Vascular Surgery, The Second Affiliated Hospital of Xi’an Jiaotong University, No. 157, Xiwu Road, 710004 Xi’an, China; 2 Department of Pathophysiology, Obesity and Diabetes Research Center, Navy Medical University, 200433 Shanghai, China

**Keywords:** Gastric cancer, EPB41L4A-AS1, Diagnosis, miR-17-5p, Progression

## Abstract

**Background:**

The incidence of gastric cancer ranks the first among digestive tract tumors in China. However, there are no specific symptoms in the early stage of the tumor and the diagnosis process is complex, so more effective detection methods are very needed. In this study, a novel long noncoding RNA (lncRNA) was introduced as a diagnostic biomarker for gastric cancer, which brought new thinking to the exploration of its pathological mechanism and clinical prediction.

**Methods:**

The level of lncRNA EPB41L4A-AS1 (EPB41L4A-AS1) in gastric cancer serum and cells was verified via real-time quantitative polymerase chain reaction (RT-qPCR). Receiver operating characteristic (ROC) curve was performed based on the EPB41L4A-AS1 level, and the diagnostic possibility of EPB41L4A-AS was analyzed. The chi-square test evaluated the correlation between EPB41L4A-AS expression and clinical information. The cells were cultured and transfected in vitro, and the mediations of abnormal EPB41L4A-AS level on the viability and motility of gastric cancer cells were verified through cell counting kit-8 (CCK-8) and Transwell assay. Furthermore, luciferase activity assay was performed to confirm the sponge molecule microRNA-17-5p (miR-17-5p) of EPB41L4A-AS1.

**Results:**

EPB41L4A-AS1 was decreased in gastric cancer, and low EPB41L4A-AS1 level indicated resultful diagnostic value. Overexpression of EPB41L4A-AS1 inhibited the activity of gastric cancer cells, while knockdown of EPB41L4A-AS1 promoted tumor deterioration. EPB41L4A-AS1 directly targeted and regulated the expression ofmiR-17-5p.

**Conclusion:**

This study elaborated that EPB41L4A-AS1 is lowly expressed in gastric cancer. Silencing EPB41L4A-AS1 was beneficial to cell proliferation, migration, and invasion. EPB41L4A-AS1 provides a new possibility for the diagnosis of gastric cancer patients by targeting miR-17-5p.

## Background

Gastric cancer starts from the gastric mucosa, mostly occurs in a single center, and may occur in any part of the stomach. More than 70% of gastric cancer cases worldwide occur in developing countries, especially in East Asia [1]. In China, an annual number of new gastric cancer patients reached 400,000, and the vast majority are diagnosed in advanced stages [2]. The etiology of gastric cancer has not been fully elucidated at present, but the investigation through domestic and foreign epidemiological has found some risk factors that may be highly correlated with the occurrence of gastric cancer, such as dietary patterns, underlying gastric diseases, and helicobacter pylori infection [3, 4]. However, the treatment of gastric cancer varies from person to person, and no systematic standard has been formed, and the prognostic effect is very general. Therefore, how to prevent the occurrence and development of gastric cancer is the focus of our research and attention.

Although long noncoding RNAs (lncRNAs) have not capable of translating proteins, they have shown functions in molecular principles and regulatory mechanisms in diseases [5]. In the discussion of gastric cancer, a large amount of evidence has revealed that lncRNAs may affect tumor progression by binding to microRNAs (miRNAs). For example, Huang et al. claimed that lncRNA TPTEP1 targeting miR-548d-3p suppressed the development of gastric cancer and may provide a new opportunity for subsequent therapy [6]. Yang et al. found that lncRNA CCAT1/miR-140-3p axis drives gastric cancer progression by increasing cell autophagy [7]. In addition, many lncRNAs have been elaborated to be associated with the diagnosis and prediction of gastric cancer [8].

EPB41L4A-AS1 is located in the chromosome 5q22.2 region, which is more than 3.8 kb in length and contains three exons [9]. In the previous literature, EPB41L4A-AS1 was described as a promising biomarker in the exploration of non-small cell lung cancer (NSCLC), chronic obstructive pulmonary disease, and type 2 diabetes [10–12]. Additionally, EPB41L4A-AS1 has regulatory capabilities in tumor metabolic reprogramming (glycolysis and glutaminolysis), which may serve as an effective target for cancer therapy [13]. However, the regulation of EPB41L4A-AS1 on gastric cancer has not been reported. Here, we examined the EPB41L4A-AS1 level in sample serum and cells, while evaluated the association of abnormal expression with clinical characteristics of patients and its diagnostic value. Furthermore, the regulation of EPB41L4A-AS1 sponge downstream miRNA on gastric cancer progression was revealed according to the cell viability and motility level. It further provides a new reference and theoretical basis for the development of diagnostic methods for gastric cancer.

## Methods

### Patient recruitment and sample acquisition

During the period from January to December 2022, blood samples were obtained from 110 gastric cancer patients recruited from The Second Affiliated Hospital of Xi’an Jiaotong University, while healthy blood samples were acquired from matched volunteers. The collected blood samples were centrifuged at 2000 r/pm for 15 min, and the extracted serum was stored in an ultra-low temperature refrigerator. The experiments mentioned were all conducted with the approval and supervision of the hospital Ethics Committee. Informed consent has been obtained from the participants involved. Under the premise of voluntary participation of all gastric cancer patients and volunteers, patients with multiple diseases, patients with adjuvant chemotherapy, and patients with cardiovascular and cerebrovascular diseases were excluded, and the clinicopathological data were recorded.

### Cell culture

Two gastric cancer cell lines (AGS and MGC-803) and gastric epithelial cell lines (GES-1) were derived from ATCC. RPMI 1640 medium was used to provide nutrients for the growth of the cells, and 10% fetal bovine serum (FBS; GE Healthcare Life Sciences, USA) was transferred at the time of configuration. The incubation environment for the cells was a humidified 37°C chamber containing 5% CO_2_.

### Transfection of cells

AGS and MGC-803 cells were transferred to culture plates and continued to incubate for about 12 h. Then, in the presence of Lipofectamine 3000 reagent (Invitrogen, USA), the overexpression plasmid (pcDNA3.1-EPB41L4A-AS1), the silencing plasmid (si-EPB41L4A-AS1) and the matched control group were transferred into AGS and MGC-803 cells, and the reconstructed cells were obtained after 48 h.

### The growth and motility properties of cells

The proliferation level of the cells was determined by CCK-8 kit (Beyotime, China). Specifically, 100 µL of the cell suspensions absorbed by a pipettes gun (Eppendorf, Germany) were inoculated into 96-well plates, followed by the addition of CCK-8 solution every 24 h, and the optical density (OD)value (450 nm) was measured after 2 h of incubation in the culture chamber. The motility ability of the cells was identified by Transwell assay. For migration assays, RPMI 1640 medium (200 µL) and cells with completed transfection were distributed above, and RPMI 1640 medium (800 µL) containing FBS was placed below. The cells that had moved below were counted after 48 h. The assessment of the cell invasion level requires the Matrigel-coated (BD Biosciences, USA) operation on the top of the system.

### Detection of PCR

RNA in sample serum and cells was extracted after the addition of TRIzol reagent (Thermo Fisher Scientific, USA), and its concentration was determined as soon as possible. Then cDNA was obtained by adding reagents on TaqMan Reverse Transcription kit (Applied Biosystems, USA) with RNA as template. SYBR Green Master Mix kit (Thermo Fisher, USA) was configured with RT-qPCR system for detection, and GAPDH and U6 were used for normalization of expression.

### Molecular mechanism study and luciferase activity assay

Bioinformatics analysis of EPB41L4A-AS1 was performed on the meridian database, and the prediction was further verified with the help of luciferase activity assays. wild type (WT)-EPB41L4A-AS1 was generated with the involvement of pmirGLO vector, and mutant type (MUT)-EPB41L4A-AS1 was constructed after mutating the binding site of EPB41L4A-AS1 and miR-17-5p. Based on the presence of Lipofectamine 3000 reagent, WT/MUT-EPB41L4A-AS1 was co-transfected into AGS cells with miR-17-5p mimic, miR-17-5p inhibitor or negative control. Finally, the luciferase activity of AGS cells was evaluated by the assay system.

### Statistical analysis

The data were processed by GraphPad Prism software 7.0 and IBM SPSS software 20.0 and displayed as mean ± standard deviation (SD). Comparison between different groups was performed by *t*-test or one-way analysis of variance. The chi-square test was used to evaluate the relationship between EPB41L4A-AS1 expression and clinicopathological features. In addition, the ROC curve demonstrated the sensitivity and specificity of EPB41L4A-AS1 in the diagnosis of gastric cancer. More than three biological replicates were performed for each set of experiments. *P* < 0.05 was statistically significant.

## Results

### EPB41L4A-AS1 is down-regulated in gastric cancer

**Fig. 1:**
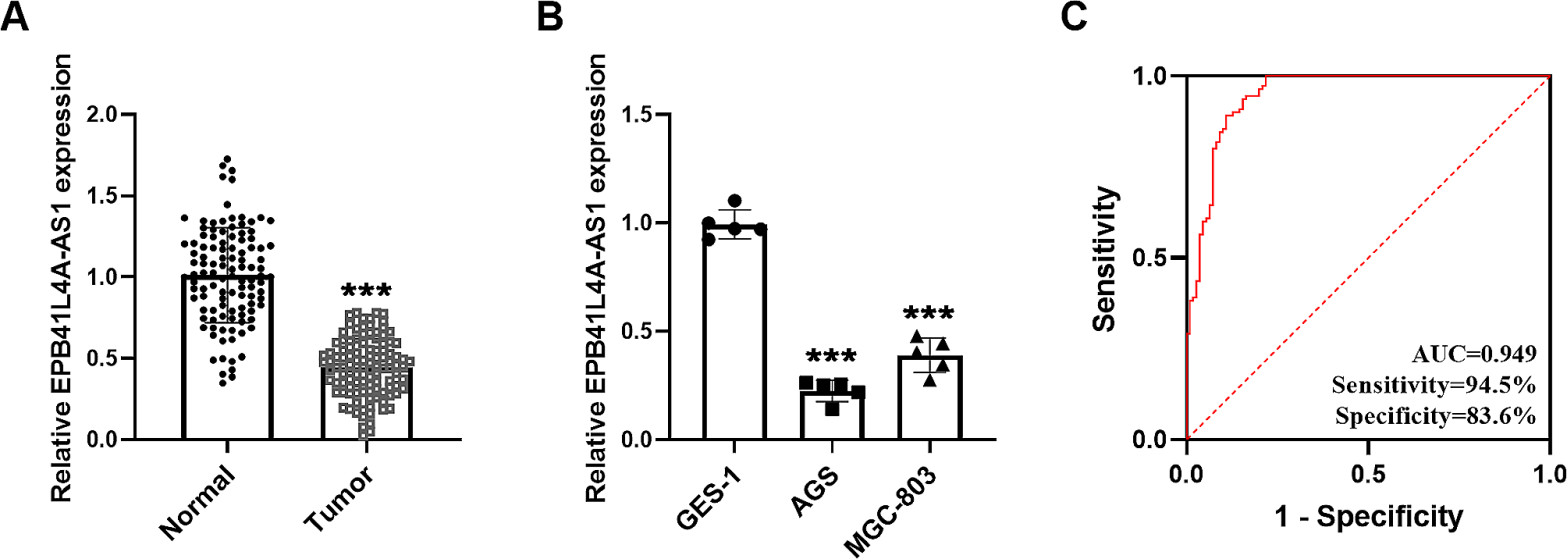
Expression and diagnostic potential of EPB41L4A-AS1. The decreased EPB41L4A-AS1 level in gastric cancer serum (**A**) and cells (**B**) was evaluated by RT-qPCR. (***P < 0.001) (**C**) EPB41L4A-AS1 had a high diagnostic value for gastric cancer (AUC = 0.949, Sensitivity = 94.5%, Specificity = 83.6%)

The decreased EPB41L4A-AS1 level in gastric cancer serum was determined by RT-qPCR in Fig. 1A. Similarly, EPB41L4A-AS1 was downregulated in gastric cancer AGS and MGC-803 cells compared with GES-1 cells (Fig. 1B). It is concluded that EPB41L4A-AS1 is negatively expressed in gastric cancer.

### Diagnostic potential and clinical relevance of EPB41L4A-AS1

The ROC curve was plotted based on EPB41L4A-AS1 expression in normal and tumor serum. As shown in Fig. 1C, the AUC was 0.949, and the high sensitivity and specificity (94.5% and 83.6%) suggested the diagnostic ability of EPB41L4A-AS1 in gastric cancer. Next, we explored the clinical relevance of EPB41L4A-AS1. In Table 1, the included gastric cancer patients were categorized into low-group (*n* = 66) and high-group (*n* = 44) by the mean value of EPB41L4A-AS1 expression. Abnormal EPB41L4A-AS1 expression was prominently associated with TNM stage (*P* = 0.024) and lymph node metastasis (*P* = 0.035) in gastric cancer patients.

**Table 1:** Relationship between EPB41L4A-AS1 expression and different clinicopathologic features in patients with gastric cancer

Indicators	Cases (*n* = 110)	EPB41L4A-AS1 expression level		*P*
		Low (*n* = 66)	High (*n* = 44)	
Age (years)				0.640
≤ 55	57	33	24	
> 55	53	33	20	
Tumor size (cm)				0.087
≤ 5	54	28	26	
> 5	56	38	18	
Sex				0.160
Female	51	27	24	
Male	59	39	20	
Differentiation				0.186
Well, Moderate	54	29	25	
Poor	56	37	19	
Lymph node metastasis				0.035
Negative	59	30	29	
Positive	51	36	15	
TNM stage				0.024
I, II	53	26	27	
III, IV	57	40	17	

### Effect of aberrant EPB41L4A-AS1 expression on the viability of gastric cancer cells

**Fig. 2:**
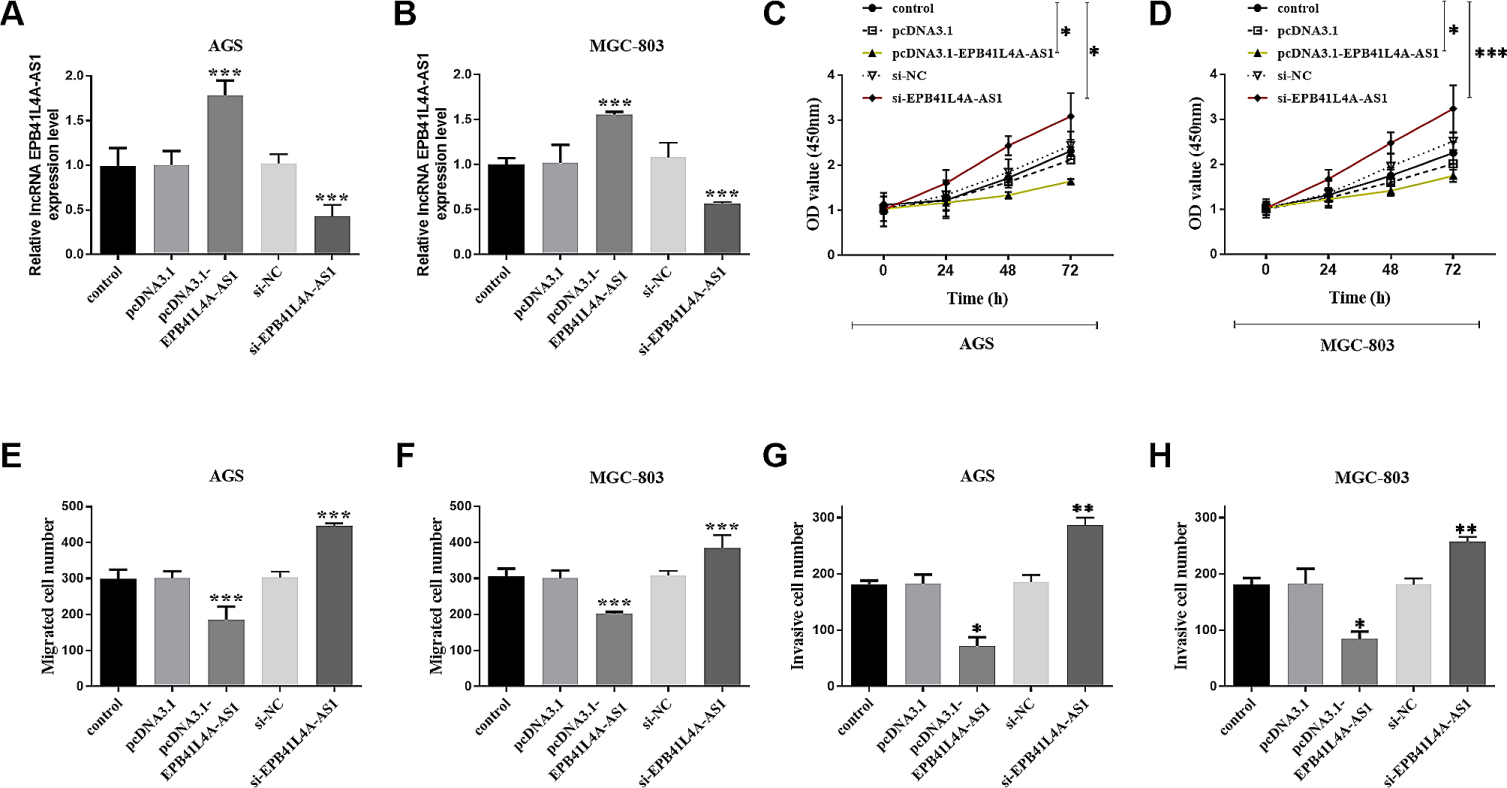
Mediation of aberrant EPB41L4A-AS1 expression to gastric cancer cells. Transfection results in AGS and MGC-803 cells (**A** and **B**). pcDNA3.1-EPB41L4A-AS1 reduced the proliferation rate (**C** and **D**), migration (**E** and **F**) and invasion (**G** and **H**) number of gastric cancer cells, while si-EPB41L4A-AS1 promoted the cell activity. (*P < 0.05, **P < 0.01, ***P < 0.001)

To clarify the influence of aberrant EPB41L4A-AS1 expression on gastric cancer progression, we designed and obtained overexpression and silencing EPB41L4A-AS1 (pcDNA3.1-EPB41L4A-AS1 and si-EPB41L4A-AS1) fragments and transfected them into AGS and MGC-803 cells, and the transfection results were shown in Fig. 2A and B. In Fig. 2C, pcDNA3.1-EPB41L4A-AS1 reduced the proliferation rate of AGS cells, while si-EPB41L4A-AS1 promoted the cell proliferation, as did MGC-803 cells (Fig. 2D**)**. In addition, pcDNA3.1-EPB41L4A-AS1 also suppressed the number of migration (Fig. 2E and F**)** and invasion (Fig. 2G and H**)**, whereas si-EPB41L4A-AS1 had the opposite effect in AGS and MGC-803 cells. These results strongly confirmed that knockdown EPB41L4A-AS1 enhanced the cell viability and motility capacity.

### EPB41L4A-AS1 targets and regulates the mir-17-5p expression

**Fig. 3:**
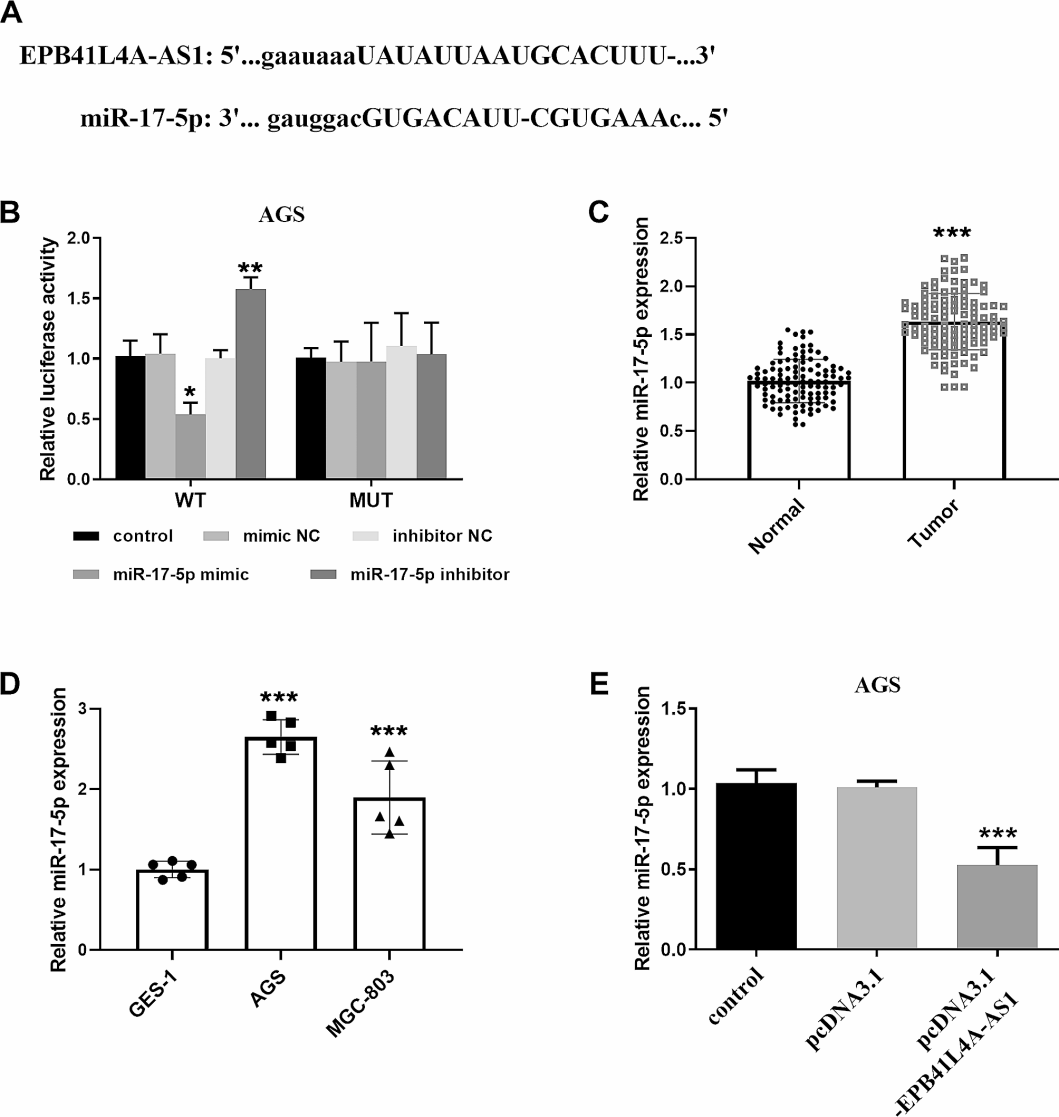
EPB41L4A-AS1 targets and regulates the miR-17-5p level. (**A**) EPB41L4A-AS1 binds to miR-17-5p and has binding targets. (**B**) EPB41L4A-AS1 directly targeted miR-17-5p. (**C**-**D**) miR-17-5p was actively expressed in gastric cancer serum and cells. (**E**) After overexpression of EPB41L4A-AS1, the miR-17-5p level in cells decreased. (*P < 0.05, ***P < 0.001)

The binding sites between EPB41L4A-AS1 and miR-17-5p were predicted by Starbase website in Fig. 3A. Therefore, we further verified the fact that EPB41L4A-AS1 sponge miR-17-5p via luciferase activity assay. Figure 3B illustrates that luciferase activity was downregulated when co-transfected with WT-EPB41L4A-AS1 and miR-17-5p mimic and upregulated when transfected with miR-17-5p inhibitor. miR-17-5p was upregulated in serum and cell samples as shown in Fig. 3C and D. Moreover, elevated EPB41L4A-AS1 inhibited the miR-17-5p expression (Fig. 3E**)**, suggesting thatEPB41L4A-AS1 had a negative regulatory effect on miR-17-5p.

**Fig. 4:**
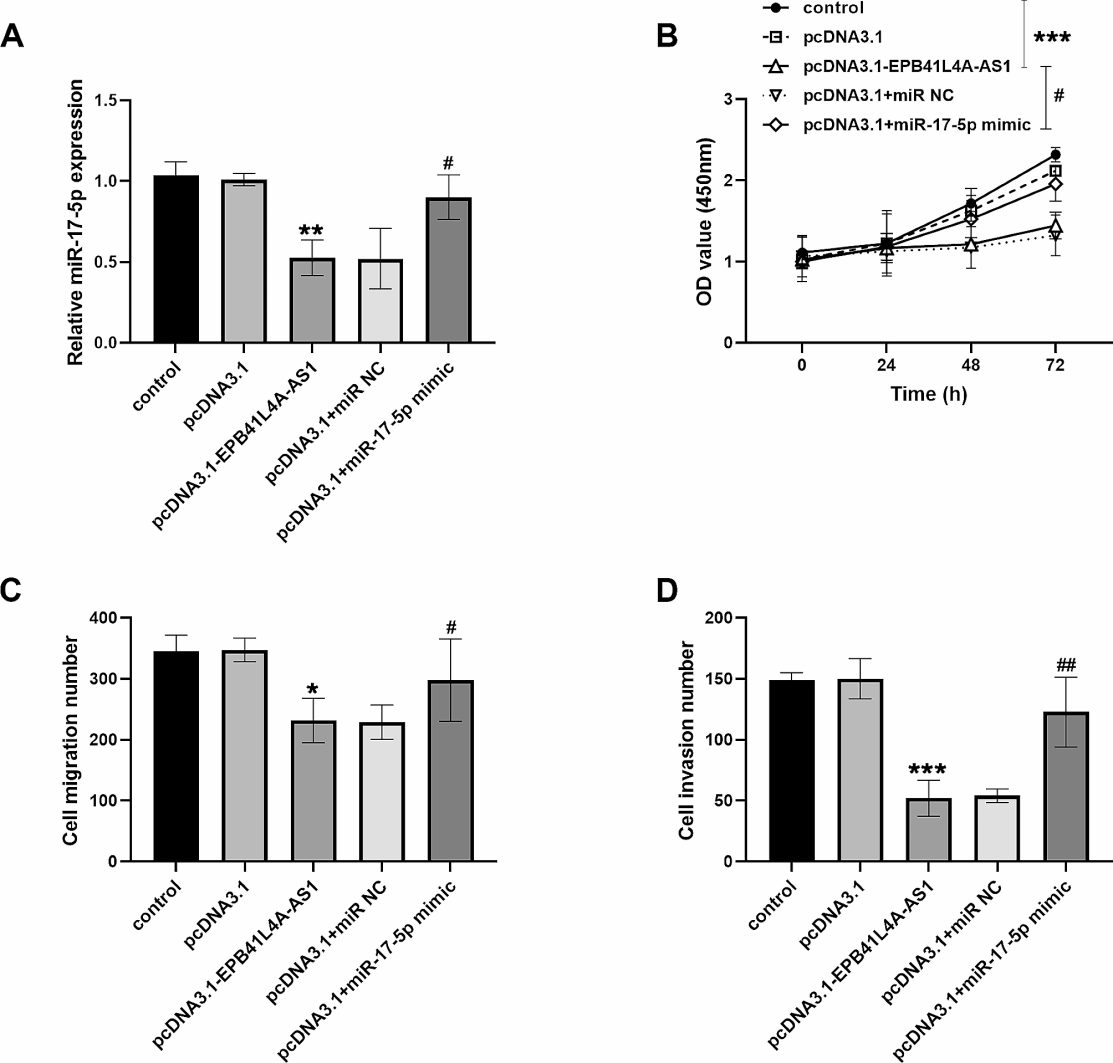
miR-17-5p mimic restored the inhibition of pcDNA3.1-EPB41L4A-AS1 on gastric cancer cell activity. (**A**) miR-17-5p content in cells after co-transfection with pcDNA3.1-EPB41L4A-AS1 and miR-17-5p mimic. (**B**-**D**) Overexpression of miR-17-5p improved the suppression of pcDNA3.1-EPB41L4A-AS1 on the biological function of gastric cancer cells. (*P < 0.05, **P < 0.01, ***P < 0.001, vs. control; ^#^*P* < 0.05, ^##^*P* < 0.01, vs. pcDNA3.1-EPB41L4A-AS1)

Overexpression of EPB41L4A-AS1 inhibited the miR-17-5p expression, while transfection with miR-17-5p mimic restored EPB41L4A-AS1 levels (Fig. 4A**)**. Similarly, co-transfection of pcDNA3.1-EPB41L4A-AS1 and miR-17-5p mimic reversed the repressive effect of EPB41L4A-AS1 upregulation on the biological functions of gastric cancer cells (Fig. 4B and D**)**.

## Discussion

Gastric cancer is a fatal tumor with high incidence, and the age of onset tends to be younger with the change of daily dietary structure [14]. In clinical application at the present stage, ultrasound gastroscopy and other related imaging examinations are generally used for early screening of patients [15], and surgical treatment, targeted therapy and chemotherapy are used for ameliorative treatment. As the early symptoms of patients are not obvious and they mainly show mild discomfort symptoms such as abdominal distension and dyspepsia, so they are often ignored as ordinary gastritis. As a result, a considerable number of patients with gastric cancer enter the middle and late stage when they are diagnosed, and their postoperative survival conditions are not ideal [16]. Therefore, it is still a serious challenge to develop a convenient and efficient prediction method for gastric cancer.

LncRNAs mediating the occurrence of various diseases have been accepted by most researchers. Through our investigation, multiple lncRNAs have been confirmed to play an effective role in the diagnosis, prognosis, and cure of gastric cancer. For example, lncRNA CRART16 promoted the formation of blood vessels in patients through the miR-122-5p/FOS axis, which can be regarded as a prognostic factor for gastric cancer [17]. LncRNA TP53TG1 was low expressed in gastric cancer, and TP53TG1 obstructed the cell cycle and process, which broaden the field of treatment for gastric cancer [18]. In addition, downregulation of lncRNA APTR has been confirmed to be closely related to the diagnosis and prognosis of gastric cancer, and knockdown of APTR was conducive to the proliferation and migration of gastric cancer cells [5]. Consistent with the previous study, EPB41L4A-AS1 was found to be a tumor suppressor and its expression was reduced both in serum samples, AGS and MGC-803 cells, which was associated with lymph node metastasis and TNM stage. In addition, EPB41L4A-AS1 was also downregulated in type 2 diabetes mellitus, Alzheimer’s disease and several cancers [19–21]. In the subsequent cell assays, overexpression of EPB41L4A-AS1 markedly inhibited the viability of AGS and MGC-803 cells, while silencing EPB41L4A-AS1 facilitated the growth and movement level of cells. This is similar to the effect of EPB41L4A-AS1 in NSCLC, and increased EPB41L4A-AS1 reduced the proliferation level of NSCLC cells [10].

Importantly, miR-17-5p was a target molecule of EPB41L4A-AS1 and was enriched in gastric cancer. miR-17-5p has been proposed as a key miRNA involved in tumor growth. It has been reported that outstanding expression of hsa_circ_0017252 may suppress the deterioration of gastric cancer through directly targets miR-17-5p [22]. Yang et al. previously indicated that miR-17-5p was elevated in colon cancer, and DNMBP-AS1 targeted miR-17-5p to inhibit tumor metastasis [23]. Positive expression of miR-17-5p that binds to lncRNAs has also been reported in rheumatoid arthritis, myocardial injury, and hepatocellular carcinoma [24–26]. In the latest literature, LINC01798/miR-17-5p axis improved the prognosis of patients with lung adenocarcinoma, and miR-17-5p level decreased after transfection of pcDNA3.1-LINC01798 [27]. This study also demonstrated that miR-17-5p expression was downregulated when EPB41L4A-AS1 was highly expressed. More deeply, silencing miR-17-5p controlled the carcinogenesis rate of gastric cancer by targeting the downstream protein PTEN, which brought new hope for patients, as confirmed by Sun et al [28]. Meanwhile, the recovery assay clarified that transfection with miR-17-5p mimic counteracted the inhibitory effect of pcDNA3.1-EPB41L4A-AS1 on the biological function of gastric cancer cells.

Inevitably, our study still has some shortcomings and regrets. First, the included sample size was limited and there were regional constraints. Secondly, there is a lack of in vivo experimental evidence, and the target gene was single. In future studies, we will increase the diversity and generalization of the sample population when possible, and further systematically explore the pathological mechanism of gastric cancer to better confirm our findings.

## Conclusions

In summary, the present study illustrated that EPB41L4A-AS1 was remarkably downregulated in gastric cancer, and knockdown EPB41L4A-AS1 accelerated the growth and motility of the cells through sponging miR-17-5p, leading to the deterioration of the tumor. EPB41L4A-AS1 has high diagnostic ability and may become a biological factor for the prediction and treatment of gastric cancer.

## Data Availability

The datasets used and/or analysed during the current study are available from the corresponding author on reasonable request.

## Abbreviations

EPB41L4A: AS1 lncRNA EPB41L4A-AS1
lncRNAs: long noncoding RNAs
miRNAs: microRNAs
NSCLC: non-small cell lung cancer
